# Metabolic Dysfunction-Associated Steatohepatitis Detected by Neutrophilic Crown-Like Structures in Morbidly Obese Patients: A Multicenter and Clinicopathological Study

**DOI:** 10.34133/research.0382

**Published:** 2024-05-29

**Authors:** Mengqi Fan, Erfei Song, Yuying Zhang, Pengfei Zhang, Bing Huang, Kaixuan Yan, Wah Yang, Subrata Chakrabarti, Hema Mahajan, Sen Yan, Ying Xu, Shuang Hua, Wei Liu, Cunchuan Wang, Aimin Xu, Dewei Ye

**Affiliations:** ^1^ Key Laboratory of Metabolic Phenotyping in Model Animals, Guangdong Pharmaceutical University, Guangzhou, China.; ^2^ Guangdong Metabolic Disease Research Center of Integrated Chinese and Western Medicine, Guangdong Pharmaceutical University, Guangzhou, China.; ^3^ Department of Bariatric Surgery, The First Affiliated Hospital of Jinan University, Guangzhou, China.; ^4^ Department of Obstetrics, Shenzhen Longhua Maternity and Child Healthcare Hospital, Shenzhen, China.; ^5^ Department of Pathology and Laboratory Medicine, Western University, London, ON, Canada.; ^6^ Institute of Clinical Pathology and Medical Research, Pathology West, NSW Health Pathology, Sydney, NSW 2145, Australia.; ^7^ Dr. Everett Chalmers Hospital, Fredericton, NB, Canada.; ^8^ School of Chinese Medicine, Guangdong Pharmaceutical University, Guangzhou, China.; ^9^ The First Affiliated Hospital of Guangzhou University of Chinese Medicine, Guangzhou, China.; ^10^ State Key Laboratory of Pharmaceutical Biotechnology, The University of Hong Kong, Hong Kong, China.; ^11^ Department of Pharmacology and Pharmacy, The University of Hong Kong, Hong Kong, China.; ^12^ Department of Medicine, The University of Hong Kong, Hong Kong, China.

## Abstract

Metabolic dysfunction-associated steatohepatitis (MASH) is the progressive form of metabolic dysfunction-associated steatotic liver disease (MASLD), and closely associated with a high risk of liver-related morbidity and mortality. Although enhanced neutrophil infiltration of the liver is a histological hallmark of MASH, the morphological pattern of hepatic neutrophils and their relevance to the definition of MASH remain unknown. This clinicopathological study aimed to determine the association of neutrophilic crown-like structures (CLSs) in liver biopsies and evaluate their relevance to the histological diagnosis of MASH. A total of 483 morbidly obese adults who underwent bariatric surgery were recruited. Neutrophilic CLSs in liver biopsies were detected by immunohistochemistry for neutrophil elastase and proteinase 3. All participants were classified into 4 histological subgroups: no MASLD (118, 24.4%), MASLD (76, 15.7%), borderline MASH (185, 38.3%), and definite MASH (104, 21.5%). In the discovery cohort (*n* = 379), the frequency of neutrophilic CLSs increased in line with the severity of liver disease. The number of neutrophilic CLSs was positively correlated with established histological characteristics of MASH. At a cutoff value of <0.3 per 20× microscopic field, the number of neutrophilic CLSs yielded a robust diagnostic accuracy to discriminate no MASLD and MASLD from borderline MASH and definite MASH; a cutoff at >1.3 per 20× microscopic field exhibited a statistically significant accuracy to distinguish definite MASH from other groups (no MASLD, MASLD, and borderline MASH). The significance of neutrophilic CLSs in identifying borderline MASH and definite MASH was confirmed in an external validation cohort (*n* = 104). The frequency of neutrophilic CLSs was significantly higher than that of macrophagic CLSs. In conclusion, neutrophilic CLSs in the liver represent a typical histological characteristic of MASH and may serve as a promising indicator to improve the diagnostic accuracy of MASH during histological assessment of liver biopsies.

## Introduction

Metabolic dysfunction-associated steatohepatitis (MASH, formerly known as non-alcoholic steatohepatitis [NASH]) is a progressive subtype of metabolic dysfunction-associated steatotic liver disease (MASLD, formerly known as non-alcoholic fatty liver disease [NAFLD]) [1,2]. There is compelling evidence from epidemiological studies that patients with MASH have a markedly increased risk of progression to liver fibrosis and, in some cases, end-stage liver disease, such as cirrhosis, liver failure, and, more rarely, hepatocellular carcinoma (HCC) [3]. Histopathological assessment of a liver biopsy remains the standard procedure to confirm a MASH diagnosis and a primary endpoint for conditional drug approval [4]. Nonetheless, marked inter-/intra-observer discordance in the histological assessment of liver biopsies based on hematoxylin and eosin as routine staining (especially in the assessment of hepatocyte ballooning, one of the key characteristics to define MASH) remains a critical issue [5,6]. Thus, poor inter- and intra-observer agreement highlights the need to identify new indicator(s) to provide additional information in the histological diagnosis of MASH.

There is accumulating evidence from murine models and humans with MASH that neutrophil infiltration of the lobular zones is markedly enhanced in the MASH liver [7]. Neutrophils have been identified in the portal zones of liver biopsies from patients with MASLD with a mixture of lymphocytes, monocytes, and occasional eosinophils [8]. Data from animal studies have demonstrated that Ly6G antibody-mediated systemic depletion of neutrophils results in marked alleviation of diet-induced MASH [9]. Although neutrophils are key players that orchestrate the overall immune response and interact with several immune cells [10], remarkably little is known about the histological patterns and the clinical relevance of hepatic infiltration of neutrophils under MASH condition.

Crown-like structures (CLSs) represent a unique histological feature and comprise a crowd of immune cells surrounding parenchymal cells in the presence of stress or severe damage to various tissues [11]. The density of CLSs has been shown to be positively correlated with adipocyte size in the visceral and subcutaneous fat of obese mice [12]. These observations suggest that macrophagic CLSs in adipose tissue are a pathological hallmark of obesity-related chronic, low-grade metabolic inflammation. Recent human studies have revealed macrophagic CLSs in breast adipose tissue to be associated with an increased risk of breast cancer, suggesting that they may also be a promising histological marker indicating high risk of breast cancer [13]. Enhanced hepatic infiltration of macrophages is well documented as a histological and pathophysiological feature of MASH [14], and CLSs characterized by CD11c-positive macrophages have been detected in the area surrounding dead or dying hepatocytes in an animal model of MASH [15]. Furthermore, the density of CLSs in human liver biopsies with MASH is positively correlated with the extent of liver fibrosis [16], suggesting that their presence in the liver is a unique histological feature of MASH.

Neutrophil elastase (NE) and proteinase 3 (PR3) are structurally related serine proteases that are stored in their active form in the azurophil granules of neutrophils [17]. Loss-of-function studies in animals have revealed NE as a key mediator in the pathogenesis of obesity-induced insulin resistance and overaccumulation of fat [18]. In patients with NAFLD and type 2 diabetes, plasma concentration of NE and PR3 is substantially increased [19]. Likewise, our previous report showed that circulating levels of NE and PR3 are increased in close association with β-cell autoimmunity in patients with type 1 diabetes [20], underscoring the potential of NE and PR3 as novel biomarkers of type 1 diabetes. Furthermore, NE-labeled neutrophils were markedly increased in the portal tract of liver biopsies from patients with NAFLD [21]. Nonetheless the histological pattern and histopathological relevance of neutrophil-derived NE and PR3 in human MASH remain largely unknown.

This study aimed to investigate the association between neutrophilic CLSs in the liver and histological features of MASH in human liver biopsies, and to evaluate the utility of neutrophilic CLSs in the detection of MASH.

## Results

### Demographics and clinicopathological characteristics

A total of 483 subjects were included in the present analysis, of whom 342 (70.8%) were female and 141 (29.2%) were male. The average age at bariatric surgery and liver biopsy was 31.3 ± 9.0 years and average body mass index (BMI) was 39.2 ± 8.0 kg/m^2^. Average blood HbA1c was 6.20% ± 1.43%, median fasting blood insulin was 18.6 mIU/l, and median homeostatic model assessment of insulin resistance (HOMA-IR) was 4.71. Average blood level of alanine aminotransferase (ALT) and aspartate aminotransferase (AST) (2 most commonly used markers of hepatocellular damage) was 51.1 ± 47.0 U/l and 31.2 ± 23.7 U/l, respectively. Histologically, patients were categorized as no MASLD (*n* = 118, 24.4%), MASLD (*n* = 76, 15.7%), borderline MASH (*n* = 185, 38.3%), or definite MASH (*n* = 104, 21.5%). Demographic and clinical characteristics assessed on the day of liver biopsy are shown in Table 1.

**Table 1.** Clinical characteristics of participants

	All subjects	Discovery cohort	Validation cohort
**Demographics**			
*n*	483	379	104
Age at biopsy, mean ± SD (years)	31.3 ± 9.0	30.9 ± 9.2	32.6 ± 8.1
Male, *n* (%)	141 (29.2)	122 (32.2)	19 (18.3)
**Anthropometric**			
Body mass index, mean ± SD (kg/m^2^)	39.2 ± 8.0	39.3 ± 8.0	38.9 ± 7.7
Waist circumference, mean ± SD (cm)	120.0 ± 17.0	120.3 ± 17.3	118.6 ± 16.0
**Hepatology panel**			
ALT, mean ± SD (U/l)	51.1 ± 47.0	51.1 ± 48.6	51.1 ± 40.8
AST, mean ± SD (U/l)	31.2 ± 23.7	31.3 ± 24.4	30.9 ± 20.7
ALP, mean ± SD (U/l)	82.1 ± 28.2	83.6 ± 29.0	76.6 ± 24.6
γ-GT, median (IQR) (U/l)	37.0 (24.0, 59.0)	36.0 (23.0, 59.0)	38.0 (25.3, 53.0)
ADA, mean ± SD (U/l)	11.9 ± 4.7	12.0 ± 4.8	11.0 ± 4.2
**Metabolic profile and other laboratory tests**			
Total cholesterol, mean ± SD (mmol/l)	5.04 ± 1.01	5.03 ± 1.00	5.12 ± 1.04
HDL, mean ± SD (mmol/l)	1.04 ± 0.23	1.04 ± 0.22	1.03 ± 0.27
LDL, mean ± SD (mmol/l)	3.04 ± 0.75	3.01 ± 0.72	3.27 ± 0.89
TG, median (IQR) (mmol/l)	1.63 (1.16, 2.22)	1.59 (1.12, 2.08)	1.87 (1.39, 2.80)
LDH, mean ± SD (U/l)	212.0 ± 73.5	204.5 ± 45.7	258.3 ± 154.4
Uric acid, median (IQR) (μmol/l)	436 (364, 511)	440 (367, 510)	420 (352, 510)
APOA, mean ± SD (g/l)	1.31 ± 0.24	1.35 ± 0.22	1.06 ± 0.23
APOB, mean ± SD (g/l)	0.97 ± 0.25	0.97 ± 0.24	0.96 ± 0.27
Fasting blood glucose, mean ± SD (mmol/l)	6.31 ± 2.46	6.30 ± 2.44	6.36 ± 2.56
HbA1c, mean ± SD (%)	6.20 ± 1.43	6.20 ± 1.46	6.13 ± 1.15
Insulin, median (IQR) (mIU/l)	18.6 (12.5, 26.8)	18.4 (12.4, 26.3)	21.0 (14.8, 32.3)
HOMA-IR, median (IQR)	4.71 (3.06, 7.28)	4.70 (3.07, 7.21)	5.05 (2.89, 7.43)
C-peptide, median (IQR) (ng/ml)	3.37 (2.54, 4.21)	3.37 (2.55, 4.22)	3.38 (2.38, 4.03)
Ferritin, mean ± SD (ng/ml)	139.22 ± 154.33	141.72 ± 158.41	108.99 ± 87.27
**Liver histological features and scores according to NASH CRN system**			
Hepatic steatosis, *n* (%)			
0 (<5%)	118 (24.4)	95 (25.1)	23 (22.1)
1 (5%–33%)	223 (46.2)	164 (43.3)	59 (56.7)
2 (34%–66%)	86 (17.8)	72 (19.0)	14 (13.5)
3 (>66%)	56 (11.6)	48 (12.7)	8 (7.7)
Lobular inflammation, *n* (%)			
0 (none)	252 (52.2)	210 (55.4)	42 (40.4)
1 (<2)	179 (37.1)	129 (34.0)	50 (48.1)
2 (2–4)	38 (7.9)	29 (7.7)	9 (8.7)
3 (>4)	14 (2.9)	11 (2.9)	3 (2.9)
Hepatocyte ballooning, *n* (%)			
0 (none)	321 (66.5)	259 (68.3)	62 (59.6)
1 (few)	143 (29.6)	102 (26.9)	41 (39.4)
2 (many)	19 (3.9)	18 (4.7)	1 (1.0)
Fibrosis, *n* (%)			
F0 (no fibrosis)	300 (62.1)	240 (63.3)	60 (57.7)
F1 (perisinusoidal or periportal fibrosis)	122 (25.3)	99 (26.1)	23 (22.1)
F2 (zone 3 perisinusoidal and periportal fibrosis)	36 (7.5)	32 (8.4)	4 (3.8)
F3 (bridging fibrosis)	20 (4.1)	7 (1.8)	13 (12.5)
F4 (cirrhosis)	5 (1.0)	1 (0.3)	4 (3.8)
**Histological subgroups, *n* (%)**			
No MASLD	118 (24.4)	95 (25.1)	23 (22.1)
MASLD	76 (15.7)	66 (17.4)	10 (9.6)
Borderline MASH	185 (38.3)	147 (38.8)	38 (36.5)
Definite MASH	104 (21.5)	71 (18.7)	33 (31.7)

ALT, alanine aminotransferase; AST, aspartate aminotransferase; ALP, alkaline phosphatase; γ-GT, gamma-glutamyl transferase; ADA, adenosine deaminase; HDL, high-density lipoprotein; LDL, low-density lipoprotein; TG, triglyceride; LDH, lactate dehydrogenase; APOA, apolipoprotein A; APOB, apolipoprotein B; HbA1c, hemoglobin A1C; HOMA-IR, homeostatic model assessment of insulin resistance.

### Severity-dependent increase in neutrophilic CLSs in liver biopsies positively correlated with biochemical and histological markers of liver lesions

**Fig. 1.**
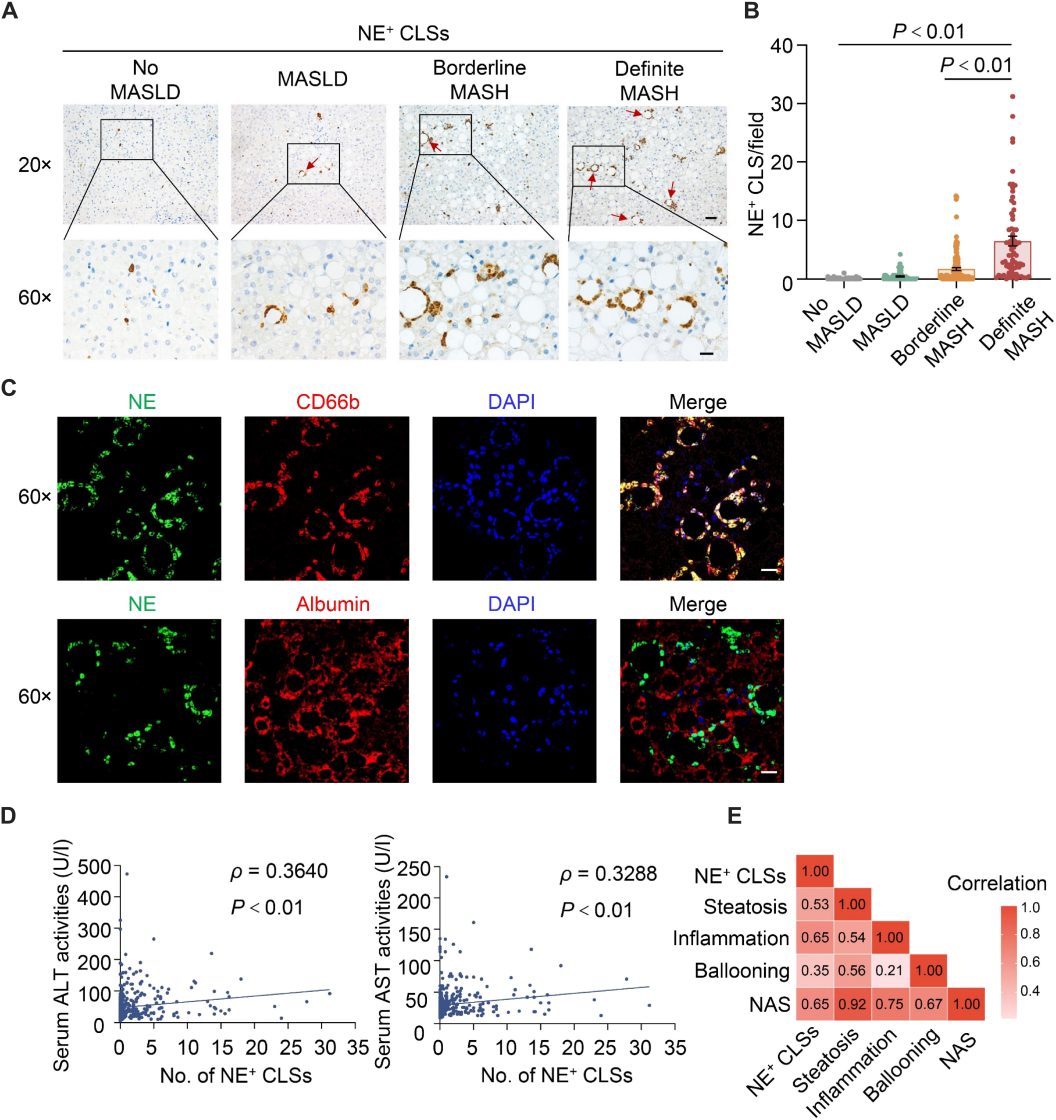
The frequency of NE-positive crown-like structures (CLS) was increased in patients with MASH and closely associated with biochemical and histological markers of MASH. Neutrophilic CLSs were detected by immunohistochemistry of neutrophil elastase (NE) in liver specimens from subjects in the discovery cohort. (A and B) Representative images of NE immunohistochemistry (A: scale bar, 50 μm in the top panel and 20 μm in the bottom panel), and semi-quantification of NE^+^ CLSs under 20× microscopic field (B). (C) Immunofluorescence of NE with co-staining of CD66b (top) and albumin (bottom) in liver biopsies with MASH (scare bar, 20 μm). (D and E) Correlation analysis of frequency of NE^+^ CLSs with serum ALT level and AST level (D), and histological features (E). Data are expressed as mean ± SEM. *P* value, correlation coefficients are indicated for the correlations.

We first measured the number of CLSs that were positive for NE or PR3 by immunohistochemistry of liver sections in the discovery cohort (379 morbidly obese subjects, 122 [32.2%] males). Histologically, 95 (25.1%) were classified as no MASLD, 66 (17.4%) as MASLD, 147 (38.8%) as borderline MASH, and 71 (18.7%) as definite MASH. The average BMI was 39.3 ± 8.0 kg/m^2^ (Table 1). Semi-quantification of NE-positive CLSs revealed a stepwise increase from no MASLD (0.05 ± 0.01) to MASLD (0.46 ± 0.10), borderline MASH (1.71 ± 0.23), and definite MASH (6.47 ± 0.83) (Fig. 1A and B). Immunofluorescence data showed that NE-positive signals markedly co-localized with neutrophil marker CD66b. NE-positive signals predominantly surrounded steatotic hepatocyte labeled by albumin (Fig. 1C). These data support neutrophils as the major cell population contributing to the formation of NE-labeled CLSs. Correlation analysis demonstrated a significant positive correlation of the frequency of NE^+^ CLSs with serum activity of ALT (*ρ =* 0.36, *P* < 0.01) and AST (*ρ* = 0.33, *P* < 0.01), the 2 most commonly used non-invasive biochemical markers of hepatocellular injury (Fig. 1D). In addition, the number of NE^+^ CLSs exhibited a significant and positive correlation with key histological features of MASH, including steatosis, lobular inflammation, hepatocyte ballooning, and total MASLD activity score (Fig. 1E).

Given that PR3 acts as a key neutrophil serine proteinase with structural and biological similarity to NE [22], we next analyzed the frequency of PR3-labeled CLSs in liver biopsies. Consistently, the frequency of CLSs detected by PR3 exhibited a disease severity-dependent increase from no MASLD (0.04 ± 0.01) to MASLD (0.38 ± 0.09), borderline MASH (1.57± 0.22), and definite MASH (6.39 ± 0.83) (Fig. S1A and B). Immunofluorescence data revealed an evident co-localization of signals with positivity of PR3 and CD66b (Fig. S1C). The number of PR3-labeled CLSs exhibited positive correlation with blood level of ALT (*ρ =* 0.34, *P* < 0.01), AST (*ρ* = 0.30, *P* < 0.01) (Fig. S1D), and histological features (Fig. S1E).

### Performance of neutrophilic CLSs in the diagnosis of MASH in the discovery cohort

**Fig. 2.**
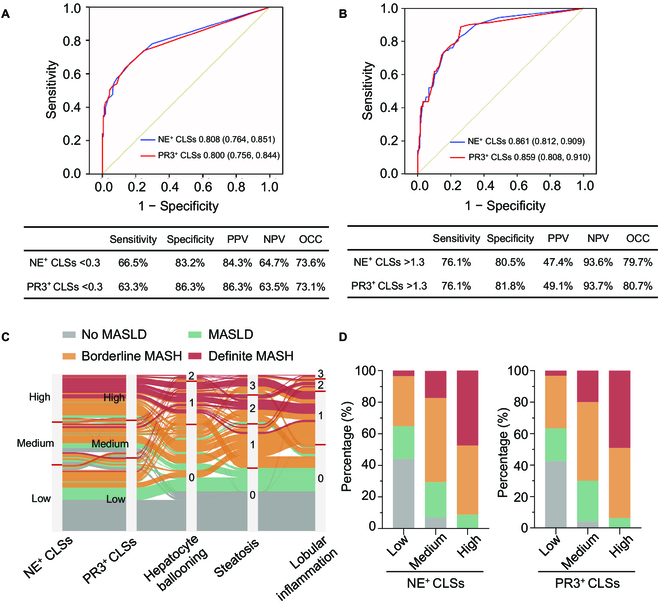
The performance of neutrophilic CLSs in the detection of MASH. (A and B) The analysis of receiver operating characteristic (ROC) curve was conducted in the discovery cohort to evaluate the potential of hepatic neutrophilic CLSs labeled by either NE or PR3 to discriminate non-MASH subtypes (no MASLD and MASLD) from MASH types (borderline MASH and definite MASH) (A), and to discriminate definite MASH from other types (B). (C) The Sankey flow diagram showing the relationship between the frequency of neutrophilic CLSs labeled by either NE or PR3, histological features of MASH, and histological subgroups. (D) The percentage of histologically diagnosed subgroups in subpopulations with low CLSs (≤0.3), medium CLSs (0.3 < CLSs ≤ 1.3), and high CLSs (>1.3) per 20× microscopic field.

To evaluate the potential of hepatic neutrophilic CLSs labeled by either NE or PR3 as a histological marker of MASH subtypes (borderline MASH and definite MASH), we conducted receiver operating characteristic (ROC) analysis in the discovery cohort. The cutoff value of neutrophilic CLSs >0.3 per 20× microscopic field yielded a robust diagnostic accuracy of AUC 0.808 (labeled with NE) or 0.800 (labeled with PR3) for identifying borderline MASH and definite MASH (Fig. 2A). Specifically, the sensitivity, specificity, positive predictive value (PPV), negative predictive value (NPV), and over correct classification (OCC) of NE-positive CLSs >0.3 per 20× microscopic field were 66.5%, 83.2%, 84.3%, 64.7%, and 73.6%, respectively; the sensitivity, specificity, PPV, NPV, and OCC corresponding to PR3-positive CLSs >0.3 per 20× microscopic field were 63.3%, 86.3%, 86.3%, 63.5%, and 73.1%, respectively (Fig. 2A).

We next assessed the capacity of neutrophilic CLSs to discriminate definite MASH from other subtypes (including no MASLD, MASLD, and borderline MASH) with the cutoff value of neutrophilic CLSs labeled by either NE or PR3 set as >1.3 per 20× microscopic field, and revealed a statistically significant diagnostic accuracy of AUC 0.861 and 0.859, respectively (Fig. 2B). The sensitivity, specificity, PPV, NPV, and OCC of NE-positive CLSs >1.3 per 20× microscopic field were 76.1%, 80.5%, 47.4%, 93.6%, and 79.7%, respectively. Meanwhile, PR3-positive CLSs with the identical cutoff value showed a sensitivity, specificity, PPV, NPV, and OCC of 76.1%, 81.8%, 49.1%, 93.7%, and 80.7%, respectively (Fig. 2B). The Sankey flow diagram demonstrates distinct distribution patterns of NE^+^ or PR3^+^ CLSs within 4 subgroups with a specific panel of histological features (Fig. 2C).

### Clinical characteristics of subjects with low or high neutrophilic CLSs

Since 0.3 and 1.3 per 20× microscopic field respectively was shown to be a significant cutoff value for NE^+^- and PR3^+^-CLSs, subjects in the discovery cohort were divided into 3 groups: low CLSs (≤0.3), medium CLSs (0.3 < CLSs ≤1.3), and high CLSs (>1.3). Subjects with high neutrophilic CLSs exhibited significantly higher parameters for obesity (BMI and waist circumference), hepatocellular injury (ALT and AST), and dysfunctional glucose metabolism (fasting blood glucose, blood insulin, HOMA-IR, and blood C-peptide) relative to those with low CLSs (Table 2). The percentage of borderline MASH and definite MASH was dramatically higher in the high CLSs group relative to those in the medium CLSs and low CLSs group (Fig. 2D and Table 2).

**Table 2.** Demographic and clinical characteristics of participants in the discovery cohort

	NE^+^ CLS levels				PR3^+^ CLS levels			
	Low	Medium	High	*P* value	Low	Medium	High	*P* value
**Demographics**								
*n*	207	58	114		219	50	110	
Male, *n* (%)	68 (32.9)	26 (44.8)	28 (24.6)	0.026	73 (33.3)	19 (38.0)	30 (27.3)	0.340
**Anthropometric**								
Body mass index, mean ± SD (kg/m^2^)	38.3 ± 7.5	40.9 ± 8.0	40.2 ± 8.9	0.035	38.3 ± 7.3	41.5 ± 8.7	40.2 ± 8.8	0.013
Waist circumference, mean ± SD (cm)	117.9 ± 16.3	124.7 ± 18.4	122.5 ± 17.9	0.008	117.9 ± 16.2	125.8 ± 18.9	122.6 ± 17.9	0.004
**History variables**								
T2DM, *n* (%)	48 (23.2)	22 (37.9)	32 (28.1)	0.077	57 (26.0)	14 (28.0)	31 (28.2)	0.902
Hypertension, *n* (%)	68 (32.9)	23 (39.7)	41 (36.0)	0.601	81 (37.0)	15 (30.0)	36 (32.7)	0.555
**Laboratory investigation**								
ALT, mean ± SD (U/l)	38.85 ± 38.33	66.19 ± 68.33	65.55 ± 47.73	<0.001	39.40 ± 37.92	70.50 ± 73.87	65.46 ± 47.10	<0.001
AST, mean ± SD (U/l)	25.64 ± 18.11	36.86 ± 34.37	38.82 ± 25.84	<0.001	25.80 ± 17.79	39.30 ± 37.44	38.69 ± 25.64	<0.001
ALP, mean ± SD (U/l)	81.91 ± 28.82	86.89 ± 33.20	84.90 ± 27.08	0.433	82.65 ± 31.21	83.18 ± 22.36	85.59 ± 27.21	0.683
γ-GT, median (IQR) (U/l)	31.0 (21.0, 55.0)	41.0 (28.0, 61.8)	39.0 (24.0, 61.0)	0.009	31.0 (21.0, 58.0)	37.9 (25.3, 58.5)	40.0 (24.0, 61.0)	0.059
ADA, mean ± SD (U/l)	11.66 ± 4.30	12.84 ± 5.66	12.14 ± 5.09	0.227	11.69 ± 4.48	13.10 ± 5.71	12.05 ± 4.86	0.169
Total cholesterol, mean ± SD (mmol/l)	4.98 ± 1.00	5.15 ± 0.96	5.05 ± 1.03	0.523	5.00 ± 1.02	5.17 ± 0.92	5.02 ± 1.01	0.556
HDL, mean ± SD (mmol/l)	1.06 ± 0.23	1.01 ± 0.19	1.02 ± 0.21	0.135	1.06 ± 0.23	1.02 ± 0.19	1.02 ± 0.21	0.164
LDL, mean ± SD (mmol/l)	2.99 ± 0.75	3.07 ± 0.68	3.02 ± 0.71	0.764	2.99 ± 0.77	3.08 ± 0.59	3.01 ± 0.69	0.761
TG, median (IQR) (mmol/l)	1.52 (1.08, 1.96)	1.71 (1.25, 2.16)	1.62 (1.28, 2.26)	0.022	1.50 (1.08, 1.96)	1.78 (1.31, 2.17)	1.63 (1.27, 2.28)	0.012
LDH, mean ± SD (U/l)	198.20 ± 42.33	209.64 ± 49.09	213.42 ± 48.33	0.011	199.67 ± 42.28	205.38 ± 50.36	213.81 ± 48.90	0.029
Uric acid, median (IQR) (μmol/l)	426 (360, 493)	453 (378, 542)	458 (381, 533)	0.090	426 (360, 493)	444 (384, 540)	462 (381, 538)	0.082
APOA, mean ± SD (g/l)	1.34 ± 0.23	1.34 ± 0.20	1.37 ± 0.20	0.504	1.34 ± 0.23	1.35 ± 0.21	1.36 ± 0.20	0.742
APOB, mean ± SD (g/l)	0.96 ± 0.25	0.99 ± 0.22	0.98 ± 0.24	0.573	0.97 ± 0.25	1.00 ± 0.21	0.98 ± 0.23	0.733
Fasting blood glucose, mean ± SD (mmol/l)	5.95 ± 1.97	6.63 ± 2.67	6.75 ± 2.95	0.01	6.11 ± 2.21	6.09 ± 1.90	6.76 ± 2.98	0.057
HbA1c, mean ± SD (%)	6.00 ± 1.32	6.56 ± 1.62	6.40 ± 1.58	0.008	6.10 ± 1.45	6.20 ± 1.08	6.41 ± 1.61	0.215
Insulin, median (IQR) (mIU/l)	16.2 (11.7, 24.4)	20.2 (13.1, 29.1)	20.7 (14.7, 27.1)	0.002	16.2 (12.0, 24.8)	19.6 (12.5, 25.7)	21.0 (15.3, 27.9)	0.003
HOMA-IR, median (IQR)	4.09 (2.72, 6.46)	5.20 (3.22, 9.13)	5.53 (4.11, 8.48)	<0.001	4.23 (2.80, 6.70)	4.62 (2.99, 7.53)	5.68 (4.19, 8.73)	<0.001
C-peptide, median (IQR) (ng/ml)	3.14 (2.26, 3.94)	3.56 (2.47, 4.59)	3.58 (2.90, 4.44)	0.001	3.15 (2.26, 4.00)	3.41 (2.55, 4.17)	3.61 (2.91, 4.46)	0.001
Ferritin, mean ± SD (ng/ml)	129.54 ± 172.74	172.80 ± 135.58	147.25 ± 140.57	0.175	132.54 ± 171.12	164.56 ± 126.66	149.38 ± 144.58	0.378
**Liver histological features and scores according to the NASH CRN system**								
Hepatic steatosis, *n* (%)				<0.001				<0.001
0 (<5%)	91 (44.0)	4 (6.9)	0 (0.0)		93 (42.5)	2 (4.0)	0 (0.0)	
1 (5%-33%)	87 (42.0)	25 (43.1)	52 (45.6)		89 (40.6)	27 (54.0)	48 (43.6)	
2 (34%-66%)	19 (9.2)	17 (29.3)	36 (31.6)		22 (10.0)	14 (28.0)	36 (32.7)	
3 (>66%)	10 (4.8)	12 (20.7)	26 (22.8)		15 (6.8)	7 (14.0)	26 (23.6)	
Lobular inflammation, *n* (%)								
0 (none)	167 (80.7)	24 (41.4)	19 (16.7)	<0.001	173 (79.0)	21 (42.0)	16 (14.5)	<0.001
1 (<2)	40 (19.3)	31 (53.4)	58 (50.9)		45 (20.5)	26 (52.0)	58 (52.7)	
2 (2-4)	0 (0.0)	2 (3.4)	27 (23.7)		1 (0.5)	1 (2.0)	27 (24.5)	
3 (>4)	0 (0.0)	1 (1.7)	10 (8.8)		0 (0.0)	2 (4.0)	9 (8.2)	
Hepatocyte ballooning, *n* (%)								
0 (none)	167 (80.7)	41 (70.7)	51 (44.7)	<0.001	178 (81.3)	34 (68.0)	47 (42.7)	<0.001
1 (few)	33 (15.9)	14 (24.1)	55 (48.2)		33 (15.1)	14 (28.0)	55 (50.0)	
2 (many)	7 (3.4)	3 (5.2)	8 (7.0)		8 (3.7)	2 (4.0)	8 (7.3)	
Fibrosis, *n* (%)								
F0 (no fibrosis)	146 (70.5)	37 (63.8)	57 (50.0)	0.028	156 (71.2)	28 (56.0)	56 (50.9)	0.005
F1 (perisinusoidal or periportal fibrosis)	46 (22.2)	13 (22.4)	40 (35.1)		47 (21.5)	13 (26.0)	39 (35.5)	
F2 (zone 3 perisinusoidal and periportal fibrosis)	11 (5.3)	6 (10.3)	15 (13.2)		12 (5.5)	6 (12.0)	14 (12.7)	
F3 (bridging fibrosis)	3 (1.4)	2 (3.4)	2 (1.8)		3 (1.4)	3 (6.0)	1 (0.9)	
F4 (cirrhosis)	1 (0.5)	0 (0.0)	0 (0.0)		1 (0.5)	0 (0.0)	0 (0.0)	
**Histological subgroups, *n* (%)**								
No MASLD	91 (44.0)	4 (6.9)	0 (0.0)	<0.001	93 (42.5)	2 (4.0)	0 (0.0)	<0.001
MASLD	43 (20.8)	13 (22.4)	10 (8.8)		46 (21.0)	13 (26.0)	7 (6.4)	
Borderline MASH	66 (31.9)	31 (53.4)	50 (43.9)		73 (33.3)	25 (50.0)	49 (44.5)	
Definite MASH	7 (3.4)	10 (17.2)	54 (47.4)		7 (3.2)	10 (20.0)	54 (49.1)	

NE, neutrophil elastase; PR3, proteinase 3; T2DM, type 2 diabetes mellitus; ALT, alanine aminotransferase; AST, aspartate aminotransferase; ALP, alkaline phosphatase; γ-GT, gamma-glutamyl transferase; ADA, adenosine deaminase; HDL, high-density lipoprotein; LDL, low-density lipoprotein; TG, triglyceride; LDH, lactate dehydrogenase; APOA, apolipoprotein A; APOB, apolipoprotein B; HbA1c, hemoglobin A1c; HOMA-IR, homeostatic model assessment of insulin resistance.

### Validation of diagnostic accuracy of neutrophilic CLSs in the validation cohort

**Fig. 3.**
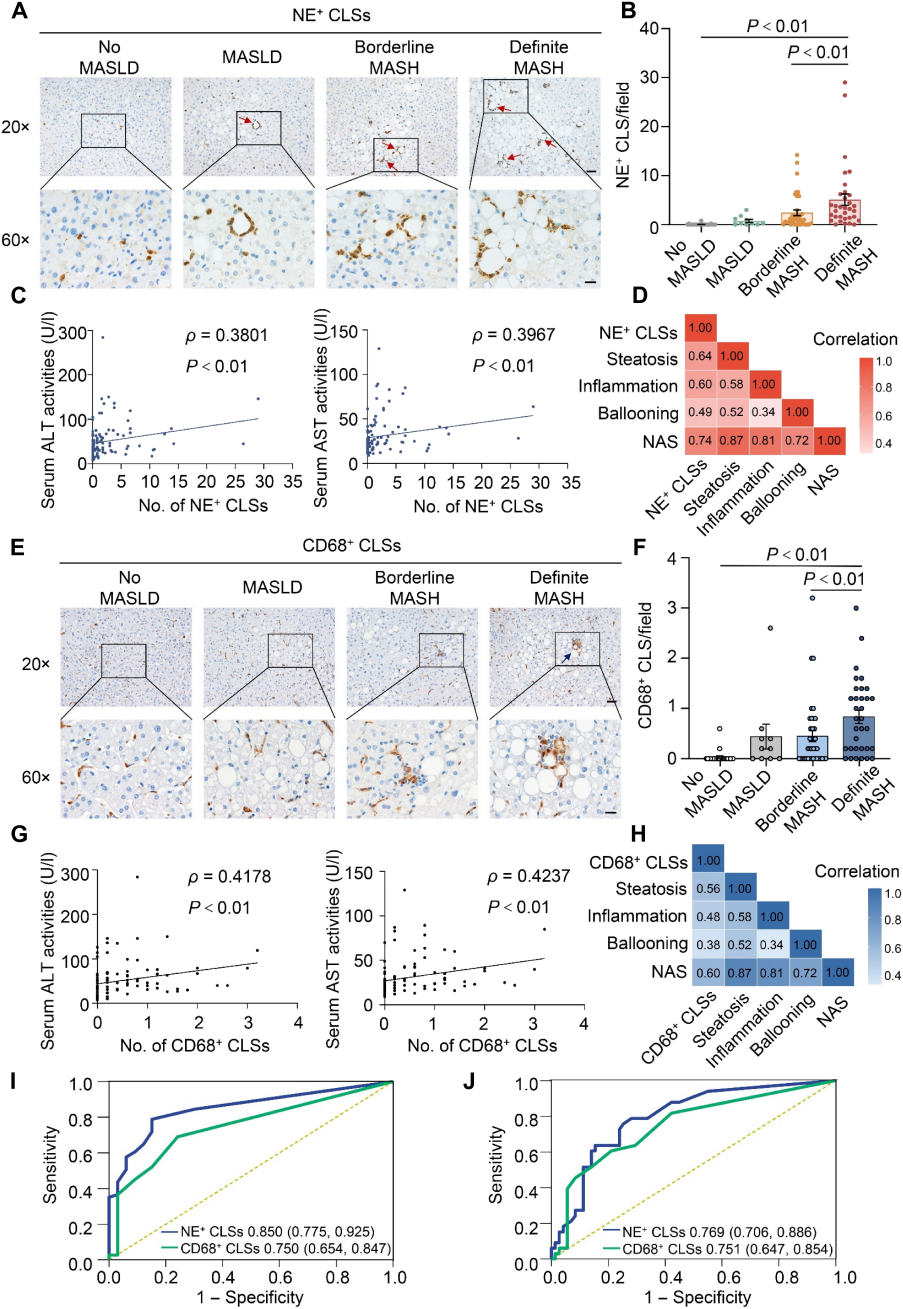
The validation of NE-labeled neutrophilic CLSs in the detection of MASH and comparison to macrophagic CLSs in external cohort. Neutrophilic CLSs were detected by immunohistochemistry of NE or macrophage marker CD68 in liver specimens from subjects in the external validation cohort (*n* = 104). (A and B) Representative images of NE immunohistochemistry (A: scale bar, 50 μm in the top panel and 20 μm in the bottom panel), and semi-quantification of NE^+^ CLSs under 20× microscopic field. (C and D) Correlation analysis of the frequency of NE^+^ CLSs and serum activity of ALT and AST (C), and histological features (D). (E and F) Representative images of CD68 immunohistochemistry in liver biopsies (E: scale bar, 50 μm in the top panel and 20 μm in the bottom panel), and semi-quantification of CD68^+^ CLSs under 20× microscopic field (F). (G and H) Correlation analysis of the frequency of CD68^+^ CLSs and serum activity of ALT and AST (G), and histological features (H). Data are expressed as mean ± SEM. (I and J) The analysis of receiver operating characteristic curve (ROC) was conducted in the external validation cohort to evaluate the potential of NE-labeled neutrophilic CLSs and CD68-labeled macrophagic CLSs in discriminating non-MASH subtypes (no MASLD and MASLD) from MASH types (borderline MASH and definite MASH) (I), and to discriminate definite MASH from other types (J).

The utility of neutrophil CLSs in histological detection of MASH was confirmed in an external validation cohort of 104 subjects, of whom 19 (18.3%) were men and average age at biopsy was 32.6 years old. Anthropometric data and blood biochemistry were comparable to those in the discovery cohort. Histologically, the number of subjects with no MASLD, MASLD, borderline MASH, and definite MASH was 23 (22.1%), 10 (9.6%), 38 (36.5%), and 33 (31.7%), respectively (Table 1). Consistent with the key characteristics identified in the discovery cohort, there was a stepwise increase in the number of neutrophilic CLSs with positivity of NE (Fig. 3A and B) or PR3 (Fig. S2A and B), from no MASLD to MASLD, borderline MASH, and definite MASH, with a significant and positive correlation with biochemical markers of liver injury (ALT and AST) and histological features of MASH (Fig. 3C and D and Fig. S2C and D). A cutoff value for neutrophilic CLSs of 0.3 and 1.3 per 20× microscopic field demonstrated reasonable diagnostic accuracy to discriminate non-MASH and definite MASH, respectively (NE^+^ CLSs shown in Fig. 3; PR3^+^ CLS shown in Fig. S2). Additionally, the pattern of anthropometric, biochemical, and histological parameters in subpopulations with low, medium, and high neutrophilic CLSs was comparable to that in the discovery cohort (Table S1).

Due to the fact that CLSs were initially defined by the formation of the clustering of macrophages surrounding adipocyte [11], and that several previous studies documented a close association between the presence of hepatic CLSs composed of macrophages and MASH [15,16], we performed immunohistochemistry of macrophage marker CD68, together with NE and PR3 in serial sections of liver biopsies with MASH. NE- or PR3-positive signals showed distinct locations from those of CD68 (Fig. S3A). These findings were further confirmed by immunofluorescence data (Fig. S3B). Thus, our data support that macrophages are not primarily involved in the formation of hepatic CLSs labeled by NE or PR3. To evaluate the diagnostic accuracy of CD68-labeled macrophagic CLSs in discriminating MASH, we performed immunohistochemistry of CD68 in liver biopsies from all participants in the validation cohort and conducted ROC analysis. The frequency of CD68-labeled macrophagic CLSs was significantly increased in the definite MASH group when compared with those in other groups (no MASLD to MASLD and borderline MASH). In addition, the number of CD68-labeled macrophagic CLSs exhibited a significant and positive correlation with markers of hepatocellular injury (ALT and AST) and histological features of MASH (Fig. 3E to H). ROC results demonstrated that CD68-labeled macrophagic CLSs exhibited suitable accuracy in discriminating MASH groups (borderline MASH, definite MASH) from non-MASH groups (no MASLD and MASLD). Of note, the performance of CD68-labeled macrophagic CLSs is slightly weaker than NE-labeled neutrophilic CLSs (Fig. 3I and J). In addition, we compared the capacity in the detection of hepatic CLSs between neutrophil serine proteases (NE and PR3) and neutrophil surface marker CD66b. The data indicated that the number of cells with a positivity of CD66b was significantly less than that of NE and PR3 (Fig. S4). Our finding is consistent with previously documented results observed in human skeletal muscles [23].

## Discussion

In this study, we investigated pathological patterns and the relevance of neutrophilic CLSs in liver biopsies from morbidly obese patients with different histological subtypes ranging from normal liver histology to MASLD (steatosis alone), borderline MASH, and MASH. The main findings of the study were as follows: (a) the number of NE- and PR3-positive CLSs was markedly increased in patients with MASH and closely associated with biochemical and histological markers of liver injury; (b) the cutoff value for neutrophilic CLSs of 0.3 and 1.3 per 20× microscopic field accurately discriminated borderline MASH and definite MASH, respectively; and (c) the diagnostic accuracy of neutrophilic CLSs was validated in an external multicenter cohort. Our data reveal that neutrophilic CLSs may represent a key pathological feature of lobular inflammation in close association with histological lesions and metabolic traits of MASH.

Although there is growing evidence that enhanced hepatic infiltration of neutrophils is a histological hallmark of MASH [7,24,25], its pathological and clinical relevance remain largely unknown. The present study revealed a severity-dependent increase in the number of NE- or PR3-positive neutrophilic CLSs in close association with well-established histological characteristics of MASH, including steatosis, lobular inflammation, and hepatocellular ballooning. Our data from ROC analysis demonstrated a slight difference in the diagnostic accuracy of neutrophilic CLSs labeled by NE or PR3. Our findings are consistent with numerous lines of evidence that the transcriptional profile of NE somehow differs to that of PR3 [26]. Furthermore, PR3 was retained intracellularly relatively efficiently, while NE was secreted to a greater extent in response to granulocyte colony-stimulating factor-induced differentiation in a human bone marrow-derived cellular myeloid differentiation model [27]. It is reasonable to speculate that neutrophils labeled by antibodies against NE or PR3 may be slightly different, although the overwhelming majority of cells labeled by NE and PR3 overlap. In the view point of histological diagnosis, NE and PR3 co-staining may promote the performance of neutrophilic CLSs for histological diagnosis of MASH.

Since dramatically increased infiltration of macrophages is well recognized as a hallmark of MASH in human [24] and rodents [28], we also detected CD68-labeled macrophagic CLSs in our study. Consistent with previous reports [15,16], our data demonstrated a close association between macrophagic CLSs and MASH. Nonetheless, CD68-labeled macrophagic CLSs are significantly lower in number than neutrophilic CLSs labeled with NE or PR3. Given that liver histology plays an extremely important role in the diagnosis of MASH, as well as preclinical and clinical evaluation of new drugs for MASH [4], our findings do suggest a possible benefit of inclusion of neutrophilic CLSs in a histopathological assessment system to improve the accuracy of a histological diagnosis of MASH. Furthermore, ballooned hepatocytes surrounded by neutrophils (also known as satellitosis) are widely recognized as a typical histological feature of alcoholic-associated liver lesions [29]. Of note, the present study recruited morbidly obese patients without excessive alcohol consumption. Therefore, our data reveal an alcohol-independent but metabolic dysfunction-dependent manner of infiltrated neutrophils in the liver in a clinical setting of MASH. This novel morphological pattern may considerably improve our understanding of pathological characteristics of MASH and highlights the need for future studies to reveal its pathophysiological relevance to MASH development.

The pathomechanism of MASLD/MASH has not been fully elucidated, but our data may provide mechanistic insight into the neutrophil-driven transition of MASLD to MASH. Previous studies of adipose tissue demonstrated that as well as being a characteristic of obesity-related adipocyte death, CLSs may function as a potent trigger of sustained metabolic inflammation in adipose tissue and whole-body insulin resistance [11]. Data from animal studies have demonstrated that neutrophils acted as key mediators to potentiate MASH pathogenesis through multiple mechanisms including enhancement of metabolic inflammation via the crosstalk with hepatic macrophages [9], boosted the release of reactive oxygen species [13], enhanced the response of stress-activated protein kinases, and increased the release of pro-inflammatory cytokines and chemokines [30,31]. Specifically, direct contact with neutrophils results in ROS enrichment in hepatocytes, thereby contributing to the development of HCC through ROS-induced lipid peroxidation and telomere damage [32]. Furthermore, co-incubation with purified human neutrophils has been shown to cause substantial cytotoxic effects on hepatocytes [33]. In line with these reports, our data on the immunohistochemistry of NE and PR3 consistently demonstrate that neutrophilic CLSs form exclusively at sites of steatotic hepatocytes in liver specimens with MASH or borderline MASH, whereas the formation of neutrophilic CLSs was rare or absent in liver specimens with MASLD or normal liver histology. Furthermore, the number of neutrophilic CLSs exhibited a positive correlation with histological score of hepatocyte ballooning and blood ALT and AST level, the most well-recognized indicators of hepatocyte death or hepatocellular damage. It is reasonable to speculate that neutrophilic CLSs surrounding steatotic hepatocytes may contribute to MASH development through direct cytotoxic effects, ROS-mediated oxidative stress, and an interplay with hepatic macrophages.

The main limitation of the current study is that all liver biopsies in the analysis were obtained from morbidly obese patients who underwent bariatric surgery. MASLD/MASH is increasingly recognized as the hepatic manifestation of a multisystem disorder with substantial heterogeneity in etiology, presentation, course, and clinical outcomes [34]. We cannot draw any conclusions about the histological patterns and pathophysiological relevance of neutrophilic CLSs in the liver of patients with lean MASH, or steatohepatitis induced by other causes (such as excessive alcohol consumption or drug hepatotoxicity). MASLD/MASH may vary with ethnicity and geographic region. Validation of our findings is warranted in larger and more diverse populations.

In conclusion, this clinicopathological correlation study revealed that the number of NE- and PR3-labeled neutrophilic CLSs was markedly increased in MASH and significantly correlated with histological lesions of liver inflammation, hepatocellular injury, and dysfunctional systemic glucose metabolism. Our results suggest that neutrophilic CLSs in the liver can serve as a severity-specific histological marker for MASH.

## Methods

### Study design and study population

This multicenter clinicopathological study recruited a total of 483 morbidly obese adults who underwent bariatric surgery from January 2017 to September 2021. Subjects in the discovery cohort (*n* = 379) were recruited from the First Affiliated Hospital of Jinan University, Guangzhou, China. Patients in the external validation cohort (*n* = 104) were recruited from (a) the Department of Hepatobiliary and Intestinal Surgery, Zhengzhou Second Hospital, Zhengzhou, China; (b) the Department of General Surgery, the Second Hospital of Anhui Medical University, Hefei, China; and (c) the Department of Gastrointestinal Surgery, General Hospital of Ningxia Medical University, Yinchuan, China. Written informed consent was obtained from all patients and control subjects. The study protocol complied with the ethical guidelines of the 1975 Declaration of Helsinki and was approved by the institutional review board at the First Affiliated Hospital of Jinan University (2016-017), Zhengzhou Second Hospital (2020-003), the Second Hospital of Anhui Medical University (YX2021-099[F1]), and the General Hospital of Ningxia Medical University (KYLL-2020-11).

### Exclusion criteria

Patients were excluded from study if they had any of the following: concomitant prescription of steatosis-inducing drugs, excessive alcohol consumption (>210 g/week in men or >140 g/week in women), infection with viral hepatitis B or C, or histological features of other concomitant chronic liver disease. Patients were also excluded if they had a diagnosis of other metabolic liver disease (hemochromatosis, α1-antitrypsin deficiency, or Wilson’s disease), autoimmune liver disease, drug-induced liver disease, liver cirrhosis-related complications (ascites, variceal bleeding, and systemic infection), HCC, and history of chronic inflammatory bowel disease, or had been prescribed antibiotics in the 2 months preceding study commencement.

### Clinical data

Demographic, serological, and radiological data were prospectively collected. Risk factors for MASLD/MASH and liver fibrosis (e.g., obesity, hypertension, hyperlipidemia, type 2 diabetes mellitus, alcohol intake, current smoking, and insufficient exercise) were recorded. Laboratory measurements included routine blood tests, liver biochemistry and full lipid profile.

### Liver biopsy and pathological diagnosis

Liver biopsies obtained from the middle of the right lobe during laparoscopic bariatric surgery for all recruited patients were processed following routine procedures. Sectioned liver biopsies were subjected to hematoxylin and eosin (H&E) staining and Masson trichrome staining. Liver slides with H&E staining and trichrome staining were independently reviewed and assessed by 3 experienced liver pathologists, Dr. Subrata Chakrabarti (Department of Pathology and Laboratory Medicine, Western University, Canada), Dr. Hema Mahajan (Department of Cytology, Western Sydney University, Australia), and Dr. Sen Yan (Dr. Everett Chalmers Hospital, Fredericton, New Brunswick, Canada), according to the NASH clinical research network (CRN) Histologic Scoring System [35], as we reported previously [36]. Pathologists were blinded to patient information. Steatosis was graded from 0 to 3, lobular inflammation was graded from 0 to 2, and hepatocyte ballooning was graded from 0 to 2. Histological diagnosis of no MAFLD, MAFLD, borderline MASH, or definite MASH was determined following the Fatty Liver Inhibition of Progression algorithm [37]. Specifically, MASH was defined as the combined presence of steatosis, hepatocyte ballooning, and lobular inflammation with or without fibrosis [38].

### Immunohistochemistry and immunofluorescence

Deparaffinized liver sections were incubated with primary antibodies against human NE (Abcam, Cat. No. ab68672, 5 μg/ml in IHC) or human PR3 (Antibody and Immunoassay Services, Hong Kong University, Cat. No. 11300, 5 μg/ml in IHC). After incubation with the REAL En-Vision Detection System (K5007, DAKO) for 15 min, slides were examined under an Olympus biological microscope BX53 and images were captured using an Olympus DP80 digital camera. Detailed information of antibodies and reagents is listed in Table S2.

### Semi-quantification of CLSs in liver biopsies

Digital images of whole slides with immunohistochemistry of PR3 or NE were captured with the Scanner 150 (Olympus). The presence of CLSs was defined when >75% of hepatocytes were encircled by PR3- or NE-positive neutrophils, following the protocol reported previously [39]. The number of CLSs was manually counted in 5 fields in each slide under 20× original magnification.

### Statistical analysis

Data are presented as mean ± standard deviation, median (interquartile range), or number (percentage) as appropriate. Differences between groups were compared using the analysis of variance test or chi-square test, referring to measurement or counted data. Spearman correlation was employed to investigate the correlation of the abundance of NE- or PR3-positive CLSs with clinical parameters. ROC analysis was performed to investigate the diagnostic value of NE and PR3 in identifying patients with borderline MASH or definite MASH. The best cutoff points were determined by the Youden’s index and the balance between sensitivity and specificity [40]. The corresponding cutoff values were validated in the validation cohort. The analysis was performed using *R* software (version 4.1.2). *P* < 0.05 (2-sided) was considered statistically significant.

## Data Availability

The data are available from the authors upon a reasonable request.

## References

[1] Rinella ME, Lazarus JV, Ratziu V, Francque SM, Sanyal AJ, Kanwal F, Romero D, Abdelmalek MF, Anstee QM, Arab JP, et al. A multisociety Delphi consensus statement on new fatty liver disease nomenclature. J Hepatol. 2023;79(6):1542–1556. 37364790 10.1016/j.jhep.2023.06.003

[2] Harrison SA, Allen AM, Dubourg J, Noureddin M, Alkhouri N. Challenges and opportunities in NASH drug development. Nat Med. 2023;29(3):562–573. 36894650 10.1038/s41591-023-02242-6

[3] Huang DQ, El-Serag HB, Loomba R. Global epidemiology of NAFLD-related HCC: Trends, predictions, risk factors and prevention. Nat Rev Gastroenterol Hepatol. 2021;18(4):223–238. 33349658 10.1038/s41575-020-00381-6 PMC8016738

[4] Bedossa P, Patel K. Biopsy and noninvasive methods to assess progression of nonalcoholic fatty liver disease. Gastroenterology. 2016;150(8):1811–1822.e1814. 27003601 10.1053/j.gastro.2016.03.008

[5] Davison BA, Harrison SA, Cotter G, Alkhouri N, Sanyal A, Edwards C, Colca JR, Iwashita J, Koch GG, Dittrich HC. Suboptimal reliability of liver biopsy evaluation has implications for randomized clinical trials. J Hepatol. 2020;73(6):1322–1332. 32610115 10.1016/j.jhep.2020.06.025

[6] Kuwashiro T, Takahashi H, Hyogo H, Ogawa Y, Imajo K, Yoneda M, Nakahara T, Oeda S, Tanaka K, Amano Y, et al. Discordant pathological diagnosis of non-alcoholic fatty liver disease: A prospective multicenter study. JGH Open. 2020;4(3):497–502. 32514460 10.1002/jgh3.12289 PMC7273711

[7] Wu L, Gao X, Guo Q, Li J, Yao J, Yan K, Xu Y, Jiang X, Ye D, Guo J. The role of neutrophils in innate immunity-driven nonalcoholic steatohepatitis: Lessons learned and future promise. Hepatol Int. 2020;14(5):652–666. 32880077 10.1007/s12072-020-10081-7

[8] Brunt EM, Kleiner DE, Wilson LA, Unalp A, Behling CE, Lavine JE, Neuschwander-Tetri BA. Portal chronic inflammation in nonalcoholic fatty liver disease (NAFLD): A histologic marker of advanced NAFLD-clinicopathologic correlations from the nonalcoholic steatohepatitis clinical research network. Hepatology. 2009;49(3):809–820. 19142989 10.1002/hep.22724 PMC2928479

[9] Ye D, Yang K, Zang S, Lin Z, Chau HT, Wang Y, Zhang J, Shi J, Xu A, Lin S, et al. Lipocalin-2 mediates non-alcoholic steatohepatitis by promoting neutrophil-macrophage crosstalk via the induction of CXCR2. J Hepatol. 2016;65(5):988–997. 27266617 10.1016/j.jhep.2016.05.041

[10] Scapini P, Cassatella MA. Social networking of human neutrophils within the immune system. Blood. 2014;124(5):710–719. 24923297 10.1182/blood-2014-03-453217

[11] Cinti S, Mitchell G, Barbatelli G, Murano I, Ceresi E, Faloia E, Wang S, Fortier M, Greenberg AS, Obin MS. Adipocyte death defines macrophage localization and function in adipose tissue of obese mice and humans. J Lipid Res. 2005;46(11):2347–2355. 16150820 10.1194/jlr.M500294-JLR200

[12] Murano I, Barbatelli G, Parisani V, Latini C, Muzzonigro G, Castellucci M, Cinti S. Dead adipocytes, detected as crown-like structures, are prevalent in visceral fat depots of genetically obese mice. J Lipid Res. 2008;49(7):1562–1568. 18390487 10.1194/jlr.M800019-JLR200

[13] Carter JM, Hoskin TL, Pena MA, Brahmbhatt R, Winham SJ, Frost MH, Stallings-Mann M, Radisky DC, Knutson KL, Visscher DW, et al. Macrophagic “crown-like structures”are associated with an increased risk of breast cancer in benign breast disease. Cancer Prev Res (Phila). 2018;11(2):113–119. 29167285 10.1158/1940-6207.CAPR-17-0245 PMC7465518

[14] Kazankov K, Jørgensen SMD, Thomsen KL, Møller HJ, Vilstrup H, George J, Schuppan D, Grønbæk H. The role of macrophages in nonalcoholic fatty liver disease and nonalcoholic steatohepatitis. Nat Rev Gastroenterol Hepatol. 2019;16(3):145–159. 30482910 10.1038/s41575-018-0082-x

[15] Itoh M, Suganami T, Kato H, Kanai S, Shirakawa I, Sakai T, Goto T, Asakawa M, Hidaka I, Sakugawa H, et al. CD11c+ resident macrophages drive hepatocyte death-triggered liver fibrosis in a murine model of nonalcoholic steatohepatitis. JCI Insight. 2017;2(22):e92902. 29202448 10.1172/jci.insight.92902 PMC5752377

[16] Itoh M, Kato H, Suganami T, Konuma K, Marumoto Y, Terai S, Sakugawa H, Kanai S, Hamaguchi M, Fukaishi T, et al. Hepatic crown-like structure: A unique histological feature in non-alcoholic steatohepatitis in mice and humans. PLoS One. 2013;8(12): Article e82163. 24349208 10.1371/journal.pone.0082163 PMC3859576

[17] Pham CT. Neutrophil serine proteases: Specific regulators of inflammation. Nat Rev Immunol. 2006;6(7):541–550. 16799473 10.1038/nri1841

[18] Talukdar S, Oh DY, Bandyopadhyay G, Li D, Xu J, McNelis J, Lu M, Li P, Yan Q, Zhu Y, et al. Neutrophils mediate insulin resistance in mice fed a high-fat diet through secreted elastase. Nat Med. 2012;18(9):1407–1412. 22863787 10.1038/nm.2885 PMC3491143

[19] Mirea AM, Toonen EJM, van den Munckhof I, Munsterman ID, Tjwa E, Jaeger M, Oosting M, Schraa K, Rutten JHW, van der Graaf M, et al. Increased proteinase 3 and neutrophil elastase plasma concentrations are associated with non-alcoholic fatty liver disease (NAFLD) and type 2 diabetes. Mol Med 2019;25(1):16. 31046673 10.1186/s10020-019-0084-3 PMC6498541

[20] Wang YD, Xiao Y, Zhong L, Ye DW, Zhang JL, Tu YT, Bornstein SR, Zhou ZG, Lam KSL, Xu AM. Increased neutrophil elastase and proteinase 3 and augmented NETosis are closely associated with β-cell autoimmunity in patients with type 1 diabetes. Diabetes. 2014;63(12):4239–4248. 25092677 10.2337/db14-0480

[21] Gadd VL, Skoien R, Fagan K, Irvine K, Powell EE, Clouston AD. The portal inflammatory infiltrate and ductular niche in non-alcoholic fatty liver disease. Hepatology. 2013;58(4):458a–458a. 24254368 10.1002/hep.26937

[22] Zimmer M, Medcalf RL, Fink TM, Mattmann C, Lichter P, Jenne DE. Three human elastase-like genes coordinately expressed in the myelomonocyte lineage are organized as a single genetic locus on 19pter. Proc Natl Acad Sci U S A. 1992;89(17):8215–8219. 1518849 10.1073/pnas.89.17.8215 PMC49888

[23] Paulsen G, Egner I, Raastad T, Reinholt F, Owe S, Lauritzen F, Brorson SH, Koskinen S. Inflammatory markers CD11b, CD16, CD66b, CD68, myeloperoxidase and neutrophil elastase in eccentric exercised human skeletal muscles. Histochem Cell Biol. 2013;139(5):691–715. 23224298 10.1007/s00418-012-1061-x

[24] Gadd VL, Skoien R, Powell EE, Fagan KJ, Winterford C, Horsfall L, Irvine K, Clouston AD. The portal inflammatory infiltrate and ductular reaction in human nonalcoholic fatty liver disease. Hepatology. 2014;59(4):1393–1405. 24254368 10.1002/hep.26937

[25] Moles A, Murphy L, Wilson CL, Chakraborty JB, Fox C, Park EJ, Mann J, Oakley F, Howarth R, Brain J, et al. A TLR2/S100A9/CXCL-2 signaling network is necessary for neutrophil recruitment in acute and chronic liver injury in the mouse. J Hepatol. 2014;60(4):782–791. 24333183 10.1016/j.jhep.2013.12.005 PMC3960359

[26] Cowland JB, Borregaard N. The individual regulation of granule protein mRNA levels during neutrophil maturation explains the heterogeneity of neutrophil granules. J Leukoc Biol. 1999;66(6):989–995. 10614782 10.1002/jlb.66.6.989

[27] Garwicz D, Lennartsson A, Jacobsen SE, Gullberg U, Lindmark A. Biosynthetic profiles of neutrophil serine proteases in a human bone marrow-derived cellular myeloid differentiation model. Haematologica. 2005;90(1):38–44. 15642667

[28] Yang S, Zhou L, Zhao T, Zhu H, Luo T, Jiang K, Shi X, Chen C, Zhang H, Zhao S, et al. Protective and adverse roles of DDX3X in different cell types in nonalcoholic steatohepatitis progression. Research (Wash D C). 2023;6:0275. 38090607 10.34133/research.0275 PMC10712874

[29] Seitz HK, Bataller R, Cortez-Pinto H, Gao B, Gual A, Lackner C, Mathurin P, Mueller S, Szabo G, Tsukamoto H. Alcoholic liver disease. Nat Rev Dis Primers. 2018;4:16. 30115921 10.1038/s41572-018-0014-7

[30] Hwang S, He Y, Xiang X, Seo W, Kim SJ, Ma J, Ren T, Park SH, Zhou Z, Feng D, et al. Interleukin-22 ameliorates neutrophil-driven nonalcoholic steatohepatitis through multiple targets. Hepatology. 2020;72(2):412–429. 31705800 10.1002/hep.31031 PMC7210045

[31] González-Terán B, Matesanz N, Nikolic I, Verdugo MA, Sreeramkumar V, Hernández-Cosido L, Mora A, Crainiciuc G, Sáiz ML, Bernardo E, et al. p38γ and p38δ reprogram liver metabolism by modulating neutrophil infiltration. Embo J. 2016;35(5):536–552. 26843485 10.15252/embj.201591857 PMC4772851

[32] Wilson CL, Jurk D, Fullard N, Banks P, Page A, Luli S, Elsharkawy AM, Gieling RG, Chakraborty JB, Fox C, et al. NFκB1 is a suppressor of neutrophil-driven hepatocellular carcinoma. Nat Commun. 2015;6:6818. 25879839 10.1038/ncomms7818 PMC4410629

[33] Marques PE, Amaral SS, Pires DA, Nogueira LL, Soriani FM, Lima BH, Lopes GA, Russo RC, Avila TV, Melgaço JG, et al. Chemokines and mitochondrial products activate neutrophils to amplify organ injury during mouse acute liver failure. Hepatology. 2012;56(5):1971–1982. 22532075 10.1002/hep.25801

[34] Eslam M, Sanyal AJ, George J. MAFLD: A consensus-driven proposed nomenclature for metabolic associated fatty liver disease. Gastroenterology. 2020;158(7):1999–2014.e1991. 32044314 10.1053/j.gastro.2019.11.312

[35] Kleiner DE, Brunt EM, Van Natta M, Behling C, Contos MJ, Cummings OW, Ferrell LD, Liu YC, Torbenson MS, Unalp-Arida A, et al. Design and validation of a histological scoring system for nonalcoholic fatty liver disease. Hepatology. 2005;41(6):1313–1321. 15915461 10.1002/hep.20701

[36] Jia X, Song E, Liu Y, Chen J, Wan P, Hu Y, Ye D, Chakrabarti S, Mahajan H, George J, et al. Identification and multicentric validation of soluble CDCP1 as a robust serological biomarker for risk stratification of NASH in obese Chinese. Cell Rep Med. 2023;4(11): Article 101257. 37918406 10.1016/j.xcrm.2023.101257 PMC10694619

[37] Bedossa P. Utility and appropriateness of the fatty liver inhibition of progression (FLIP) algorithm and steatosis, activity, and fibrosis (SAF) score in the evaluation of biopsies of nonalcoholic fatty liver disease. Hepatology. 2014;60(2):565–575. 24753132 10.1002/hep.27173

[38] Bedossa P, Poitou C, Veyrie N, Bouillot JL, Basdevant A, Paradis V, Tordjman J, Clement K. Histopathological algorithm and scoring system for evaluation of liver lesions in morbidly obese patients. Hepatology. 2012;56(5):1751–1759. 22707395 10.1002/hep.25889

[39] Maliniak ML, Cheriyan AM, Sherman ME, Liu Y, Gogineni K, Liu J, He J, Krishnamurti U, Miller-Kleinhenz J, Ashiqueali R, et al. Detection of crown-like structures in breast adipose tissue and clinical outcomes among African-American and White women with breast cancer. Breast Cancer Res. 2020;22(1):65. 32552729 10.1186/s13058-020-01308-4 PMC7298873

[40] Perkins NJ, Schisterman EF. The inconsistency of “optimal” cutpoints obtained using two criteria based on the receiver operating characteristic curve. Am J Epidemiol. 2006;163(7):670–675. 16410346 10.1093/aje/kwj063 PMC1444894

