# Supplementary material for: Metabolic Dysfunction-Associated Steatohepatitis Detected by Neutrophilic Crown-Like Structures in Morbidly Obese Patients: A Multicenter and Clinicopathological Study
Source: Research (Wash D C). 2024 May 29;7:0382. doi: 10.34133/research.0382 (PMC11134285; doi:10.34133/research.0382)
Supplement: Supplementary 1 — Figs. S1 to S4 Tables S1 and S2 [file research.0382.f1.pdf]

Legends for Supplementary Figures

Supplementary Figure 1. The frequency of PR3-positive crown-like structures (CLS) was increased in MASH liver in close association with biochemical and histological markers of MASH in subjects in discovery cohort

Neutrophilic CLSs were detected by immunohistochemistry of proteinase 3 (PR3) in liver specimens from subjects in the discovery cohort. (**A, B**) Representative images of PR3 immunohistochemistry (A, scale bar 50 μm in upper panel, 20 μm in lower panel), and semi-quantification of $PR3^+$ CLSs under 20× microscopic field (B). (**C**) Immunofluorescence of NE with co-staining of CD66b (upper) and albumin (lower) in liver biopsies with MASH (scare bar, 20 μm). (**D, E**) Correlation analysis of frequency of $PR3^+$ CLSs with serum activity of ALT, AST (D), and histological features (E). Data are expressed as mean ± SEM. *P* value and correlation coefficients are indicated.

Supplementary Figure 2. The frequency of PR3-positive crown-like structures (CLS) in liver tissue and correlation analysis in the external validation cohort

Neutrophilic CLSs were detected by immunohistochemistry of either neutrophil elastase or proteinase 3 (PR3) in liver specimens from subjects in the external validation cohort (n = 104). (**A, B**) Representative images of PR3 immunohistochemistry (A, scale bar 50 μm in upper panel, 20 μm in lower panel), and semi-quantification of $PR3^+$ CLSs under 20× microscopic field. (**C, D**) Correlation analysis of the frequency of $PR3^+$ CLSs and serum activity of ALT, AST, and histological features (D). (**E, F**) The analysis of operating characteristic (ROC) was conducted in the external validation cohort to evaluate the performance of PR3-positive neutrophilic CLSs to discriminate non-MASH subtypes (no MASLD, MASLD) from MASH types (borderline MASH and definite MASH) (E), and to discriminate definite MASH from other types (F).

Supplementary Figure 3. Immunostaining reveals different patterns between macrophage marker CD68 and neutrophil serine proteases in MASH liver biopsies.

Liver biopsies from participants in the validation cohort were used for immunohistochemistry and immunofluorescence staining. (**A)** Representative images of immunohistochemistry of CD68, NE, and PR3 in serial sections of liver biopsies from 3 MASH patients. Red closed arrow indicates landmark structure. Scale bar, 50

µm in 20× magnification fields (left) and 20 µm in 60× magnification fields (right). (**B)** Immunofluorescence of CD68 with co-staining of NE (upper), and PR3 (lower) in liver biopsies with MASH (scale bar, 20 µm).

Supplementary Figure 4. Immunohistochemistry of CD66b, NE, and PR3 in serial sections of liver biopsies with MASH

Immunohistochemistry of CD66b, NE, and PR3 was performed in liver biopsies from participants in the validation cohort. (**A**) Key information in immunohistochemistry protocol. (**B**) Representative images of immunostaining of CD68, NE, and PR3 in serial sections of liver biopsies with MASH (scale bar 50 µm in 20× magnification fields, 20 µm in 60× magnification fields). Red closed arrow indicates landmark structure for serial sections.

Supplementary Figure 1

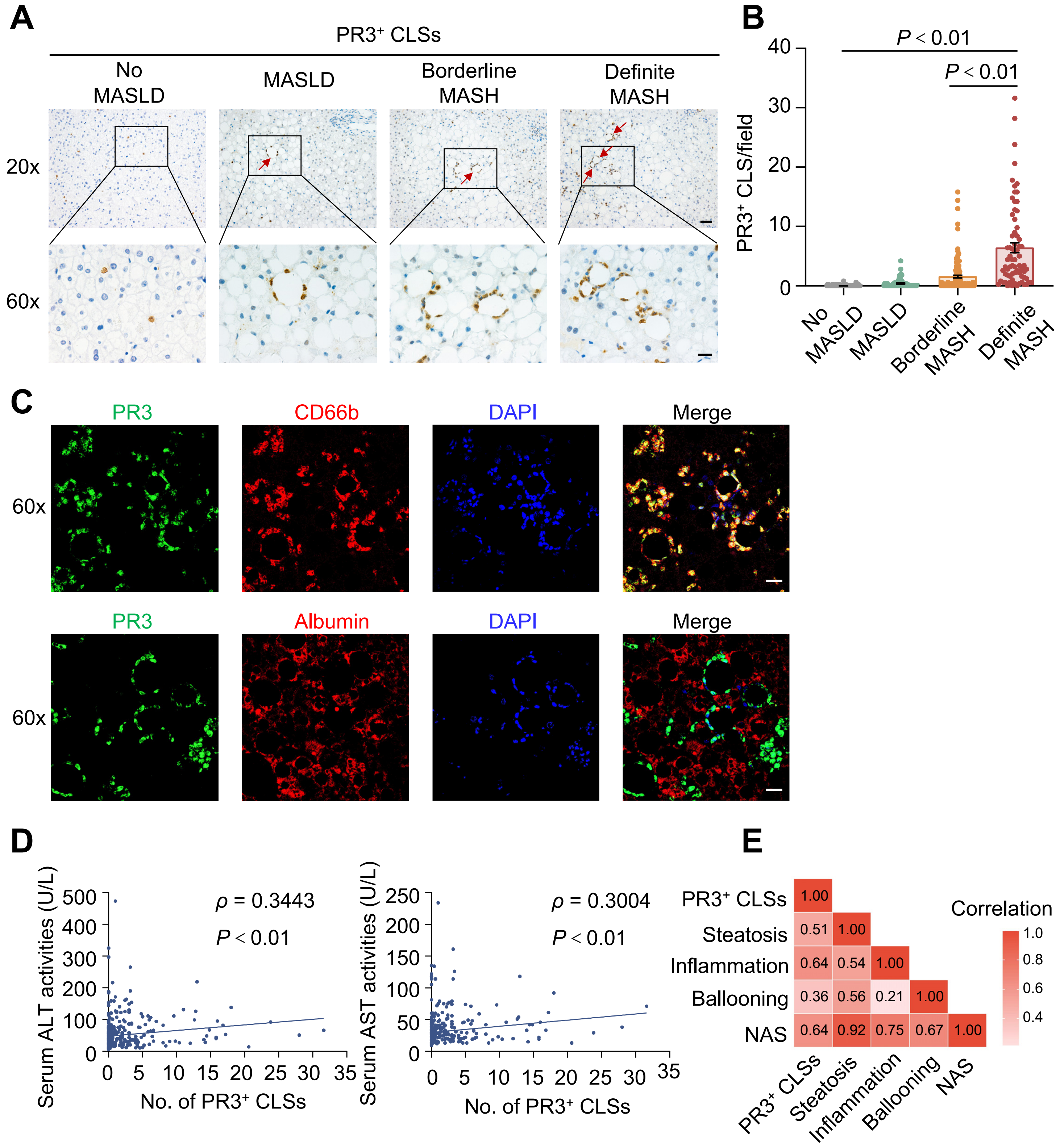

Supplementary Figure 2

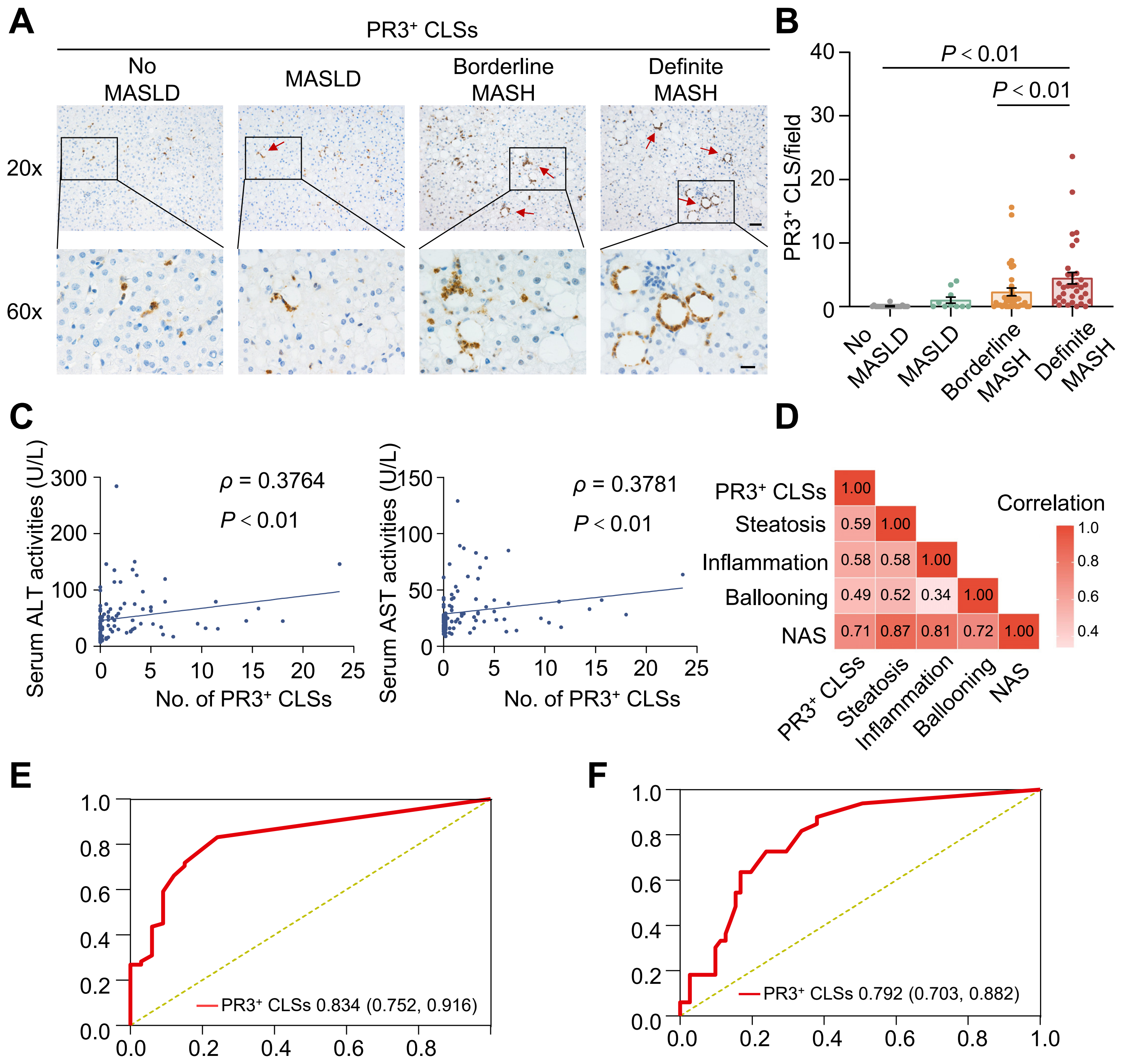

	Sensitivity	Specificity	PPV	NPV	OCC
PR3⁺ CLSs <0.3	71.8%	84.8%	91.1%	58.3%	76.0%

	Sensitivity	Specificity	PPV	NPV	OCC
PR3⁺ CLSs >1.3	72.7%	76.1%	58.5%	85.7%	75.0%

Supplementary Figure 3

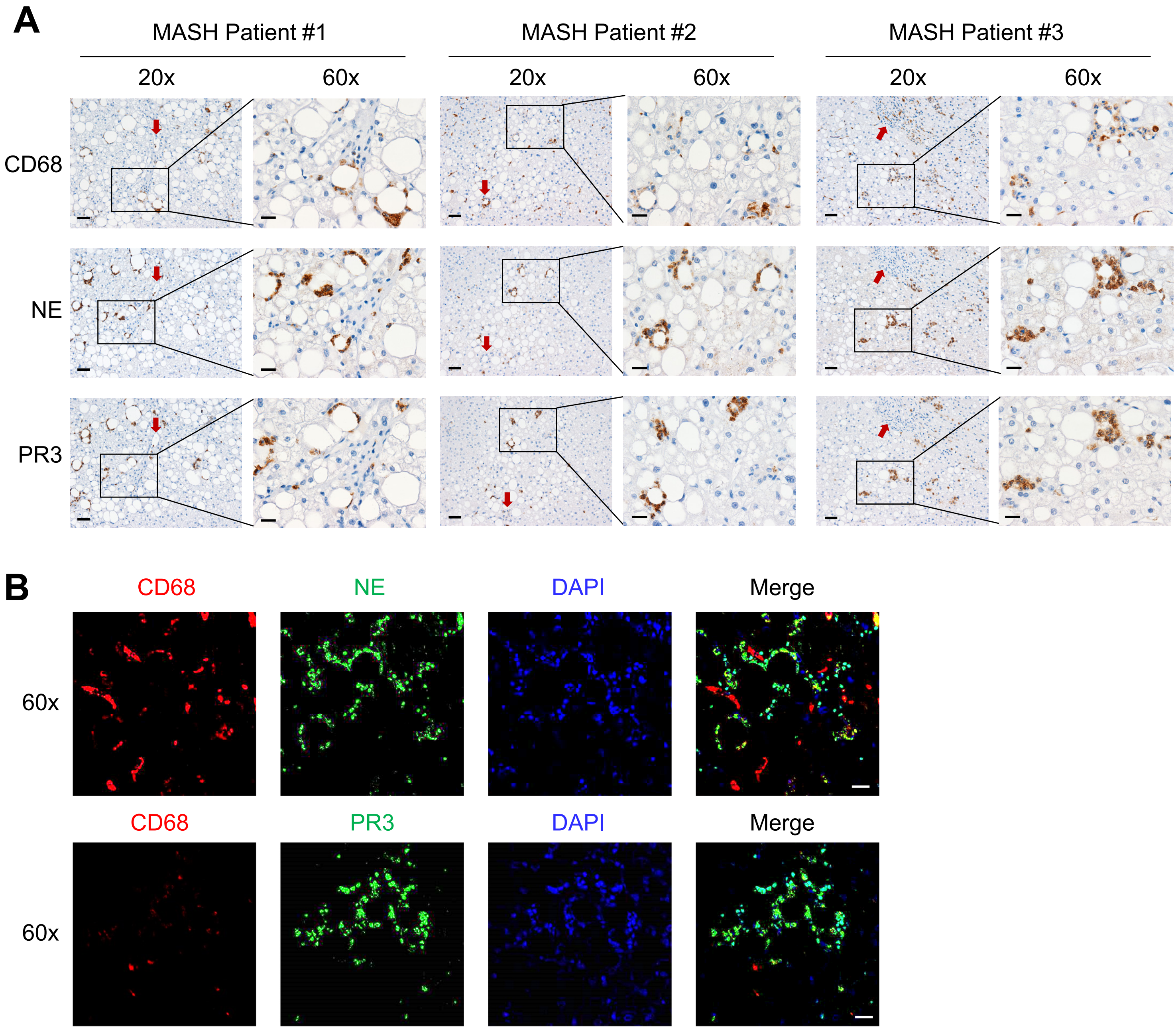

Supplementary Figure 4

A

	Primary antibodies			Secondary antibodies		Chromogen incubation
	Company	Cat. *No*.	Working conc	Company	Cat. *No.*	
**CD66b**	BioLegend	305102	5 ug/ml	DAKO	K5007	3 min
**NE**	Abcam	ab68672	5 ug/ml	DAKO	K5007	1 min
**PR3**	AIS Hong Kong	11300	5 ug/ml	DAKO	K5007	1 min

B

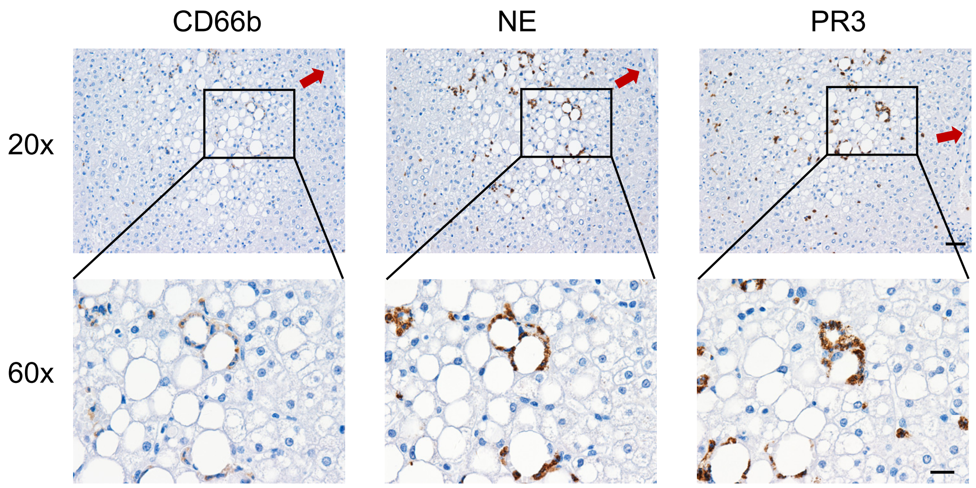

Supplementary Table 1. Demographic and clinical characteristics of participants in validation cohort

	NE⁺ CLS levels				PR3⁺ CLS levels			
	Low	Median	High	*P* value	Low	Median	High	*P* value
**Demographics**								
No.	43	18	43		48	15	41	
Male, No. (%)	7 (16.3)	4 (22.2)	8 (18.6)	0.858	8 (16.7)	4 (26.7)	7 (17.1)	0.663
**Anthropometric**								
Body mass index, mean ± SD, kg/m^2	36.77 ± 6.08	37.00 ± 6.29	41.66 ± 8.74	0.011	36.49 ± 6.09	36.57 ± 5.05	42.40 ± 8.77	0.001
Waist circumference, mean ± SD, cm	113.47 ± 13.29	114.94 ± 10.83	124.84 ± 18.15	0.005	112.84 ± 13.01	114.86 ± 9.05	126.33 ± 18.02	<0.001
**Laboratory investigation**								
ALT, mean ± SD, U/L	35.86 ± 22.67	41.72 ± 25.96	70.37 ± 51.43	<0.001	35.15 ± 21.89	49.60 ± 32.93	70.42 ± 51.59	<0.001
AST, mean ± SD, U/L	22.24 ± 9.59	25.85 ± 12.09	41.74 ± 26.43	<0.001	21.98 ± 9.51	32.29 ± 17.25	40.88 ± 26.49	<0.001
ALP, mean ± SD, U/L	74.36 ± 21.42	79.36 ± 34.21	77.81 ± 23.08	0.714	74.11 ± 23.31	80.25 ± 34.16	78.38 ± 22.40	0.608
γ-GT, median (IQR), U/L	29.0 (19.5, 42.0)	40.5 (25.0, 57.8)	47.1 (35.0, 62.0)	0.001	28.5 (20.0, 43.3)	34.0 (24.3, 59.8)	47.6 (35.8, 63.3)	<0.001
ADA, mean ± SD, U/L	9.75 ± 2.89	11.17 ± 4.63	12.50 ± 5.00	0.098	9.79 ± 2.88	12.00 ± 5.89	12.50 ± 4.87	0.070
Total cholesterol, mean ± SD, mmol/L	4.99 ± 1.02	5.11 ± 1.02	5.25 ± 1.09	0.65	5.01 ± 1.03	5.15 ± 0.96	5.24 ± 1.09	0.709
HDL, mean ± SD, mmol/L	1.10 ± 0.23	1.02 ± 0.25	0.97 ± 0.33	0.291	1.09 ± 0.24	0.98 ± 0.16	0.98 ± 0.33	0.340
LDL, mean ± SD, mmol/L	3.34 ± 0.74	3.22 ± 0.69	3.23 ± 1.13	0.911	3.28 ± 0.73	3.42 ± 0.62	3.20 ± 1.14	0.854
TG, median (IQR), mmol/L	1.63 (1.19, 2.19)	2.60 (1.40, 3.39)	2.15 (1.69, 3.08)	0.007	1.62 (1.20, 2.23)	2.63 (1.57, 3.39)	2.16 (1.71, 3.08)	0.005
LDH, mean ± SD, U/L	231.83 ± 134.83	210.67 ± 103.45	296.36 ± 177.26	0.197	223.93 ± 126.18	226.29 ± 118.57	304.00 ± 181.22	0.138
Uric acid, median (IQR), μmol/L	415 (308, 485)	380 (366, 513)	439 (386, 523)	0.389	404 (313, 477)	421 (379, 532)	438 (381, 516)	0.286
APOA, mean ± SD, g/L	1.03 ± 0.19	1.06 ± 0.24	1.08 ± 0.27	0.777	1.04 ± 0.21	1.08 ± 0.20	1.08 ± 0.27	0.772

APOB, mean ± SD, g/L	0.95 ± 0.23	0.94 ± 0.36	0.99 ± 0.26	0.818	0.92 ± 0.27	1.05 ± 0.23	1.00 ± 0.27	0.397
Fasting blood glucose, mean ± SD, mmol/L	5.59 ± 1.90	6.70 ± 3.54	7.00 ± 2.50	0.031	5.68 ± 1.96	6.75 ± 3.71	7.01 ± 2.55	0.041
HbA1c, mean ± SD, %	5.94 ± 1.43	5.77 ± 0.43	6.40 ± 0.96	0.323	5.96 ± 1.43	5.64 ± 0.38	6.45 ± 0.96	0.202
Insulin, median (IQR), mU/L	16.9 (11.1, 27.5)	19.1 (14.6, 28.7)	25.5 (18.6, 36.2)	0.174	16.9 (11.1, 28.5)	16.9 (10.9, 25.0)	26.0 (19.2, 37.2)	0.079
HOMA-IR, median (IQR)	3.49 (2.37, 5.83)	4.22 (2.18, 6.68)	6.16 (4.72, 11.14)	0.027	3.49 (2.37, 6.63)	4.22 (2.42, 5.53)	6.16 (4.72, 11.14)	0.040
C-peptide, median (IQR), ng/mL	3.18 (2.58, 4.08)	1.74 (1.74, 1.74)	3.58 (2.79, 3.88)	0.356	3.18 (2.58, 4.08)	1.74 (1.74, 1.74)	3.58 (2.79, 3.88)	0.356
Ferritin, mean ± SD, ng/mL	83.00 ± 64.203	102.00 ± 104.31	149.57 ± 98.12	0.182	99.31 ± 81.12	38.67 ± 31.21	152.60 ± 93.65	0.066
**Liver histological features and scores according to NASH CRN system**								
Hepatic steatosis, No. (%)				<0.001				
0 (<5%)	22 (51.2)	1 (5.6)	0 (0.0)		22 (45.8)	1 (6.7)	0 (0.0)	<0.001
1 (5%-33%)	19 (44.2)	13 (72.2)	27 (62.8)		22 (45.8)	11 (73.3)	26 (63.4)	
2 (34%-66%)	2 (4.7)	4 (22.2)	8 (18.6)		4 (8.3)	2 (13.3)	8 (19.5)	
3 (>66%)	0 (0.0)	0 (0.0)	8 (18.6)		0 (0.0)	1 (6.7)	7 (17.1)	
Lobular inflammation, No. (%)								
0 (none)	30 (69.8)	6 (33.3)	6 (14.0)	<0.001	30 (62.5)	5 (33.3)	7 (17.1)	<0.001
1 (<2)	13 (30.2)	12 (66.7)	25 (58.1)		18 (37.5)	9 (60.0)	23 (56.1)	
2 (2-4)	0 (0.0)	0 (0.0)	9 (20.9)		0 (0.0)	1 (6.7)	8 (19.5)	
3 (>4)	0 (0.0)	0 (0.0)	3 (7.0)		0 (0.0)	0 (0.0)	3 (7.3)	
Hepatocyte ballooning, No. (%)								
0 (none)	37 (86.0)	11 (61.1)	14 (32.6)	<0.001	42 (87.5)	7 (46.7)	13 (31.7)	<0.001
1 (few)	6 (14.0)	7 (38.9)	28 (65.1)		6 (12.5)	8 (53.3)	27 (65.9)	
2 (many)	0 (0.0)	0 (0.0)	1 (2.3)		0 (0.0)	0 (0.0)	1 (2.4)	
Fibrosis, No. (%)								

F0 (no fibrosis)	35 (81.4)	8 (44.4)	17 (39.5)	0.001	37 (77.1)	6 (40.0)	17 (41.5)	0.011
F1 (perisinusoidal or periportal fibrosis)	4 (9.3)	7 (38.9)	12 (27.9)		5 (10.4)	6 (40.0)	12 (29.3)	
F2 (zone 3 perisinusoidal and periportal fibrosis)	1 (2.3)	2 (11.1)	1 (2.3)		2 (4.2)	1 (6.7)	1 (2.4)	
F3 (bridging fibrosis)	3 (7.0)	1 (5.6)	9 (20.9)		4 (8.3)	2 (13.3)	7 (17.1)	
F4 (cirrhosis)	0 (0.0)	0 (0.0)	4 (9.3)		0 (0.0)	0 (0.0)	4 (9.8)	
**Histological subgroups, No. (%)**								
No MASLD	22 (51.2)	1 (5.6)	0 (0.0)	<0.001	22 (45.8)	1 (6.7)	0 (0.0)	<0.001
MASLD	6 (14.0)	2 (11.1)	2 (4.7)		6 (12.5)	1 (6.7)	3 (7.3)	
Borderline MASH	11 (25.6)	11 (61.1)	16 (37.2)		16 (33.3)	8 (53.3)	14 (34.1)	
Definite MASH	4 (9.3)	4 (22.2)	25 (58.1)		4 (8.3)	5 (33.3)	24 (58.5)	

Abbreviations: **NE**, neutrophil elastase; **PR3**, proteinase 3; **ALT**, alanine aminotransferase; **AST**, aspartate aminotransferase; **ALP**, alkaline phosphatase; **γ-GT**, gamma-glutamyl transferase; **ADA**, adenosine deaminase; **HDL**, high density lipoprotein; **LDL**, low density lipoprotein; **TG**, triglyceride; **LDH**, lactate dehydrogenase; **APOA**, apolipoprotein A; **APOB**, apolipoprotein B; **HbA1c**, hemoglobin A1c; **HOMA-IR**, homeostatic model assessment of insulin resistance.

Supplementary Table 2. Key resources table

REAGENT or RESOURCE	SOURCE	IDENTIFIER
**Antibodies**		
Rabbit anti-NE	Abcam	Cat# ab68672; RRID: AB_1658868 (5 µg/mL in IHC and IF)
Rabbit anti-human PR3	Antibody and Immunoassay Services, HKU	Cat# 11300 (5 µg/mL in IHC and IF)
Mouse anti-CD68	Abcam	Cat# ab955; RRID: AB_307338 (0.371 µg/mL in IHC; 22.240 µg/mL in IF)
Mouse anti-human CD66b	BioLegend	Cat# 305102; RRID: AB_314494 (5 µg/mL in IHC; 10 µg/mL in IF)
Goat anti-Albumin	R&D Systems	Cat# AF3329 (5 µg/mL in IF)
REAL EnVision Detection System	DAKO	Cat# K5007; RRID: AB_2888627
REAL EnVision Detection System	DAKO	Cat# K4001; RRID: AB_2827819
Goat anti-rabbit Alexa Fluor 488	Thermo Fisher Scientific	Cat# A32731; RRID: AB_2633280 (10 µg/mL in IF)
Donkey anti-rabbit Alexa Fluor 488	Thermo Fisher Scientific	Cat# A21206; RRID: AB_2535792 (10 µg/mL in IF)
Donkey anti-mouse Alexa Fluor 594	Thermo Fisher Scientific	Cat# A21203; RRID: AB_2535789 (10 µg/mL in IF)
Donkey anti-goat Alexa Fluor 594	Thermo Fisher Scientific	Cat# A11058; RRID: AB_2534105 (10 µg/mL in IF)
**Chemicals, peptides, and recombinant proteins**		
DAPI Fluoromount-G™	Yeasen Biotechnology	Cat# 36308ES20

Abbreviations: **IHC**, immunohistochemistry; **IF**, immunofluorescence.
